# Concurrent validity of a low‐cost and time‐efficient clinical sensory test battery to evaluate somatosensory dysfunction

**DOI:** 10.1002/ejp.1456

**Published:** 2019-08-28

**Authors:** Guan Cheng Zhu, Karina Böttger, Helen Slater, Chad Cook, Scott F. Farrell, Louise Hailey, Brigitte Tampin, Annina B. Schmid

**Affiliations:** ^1^ Nuffield Department of Clinical Neurosciences University of Oxford Oxford UK; ^2^ Institute of Allied Health Sciences, College of Medicine National Cheng Kung University Tainan Taiwan (R.O.C.); ^3^ Centre of Pain Medicine Swiss Paraplegic Centre Nottwil Switzerland; ^4^ School of Physiotherapy and Exercise Science Curtin University Perth WA Australia; ^5^ Department of Orthopaedics, Duke Clinical Research Institute Duke University Durham NC USA; ^6^ RECOVER Injury Research Centre NHMRC Centre for Research Excellence in Recovery Following Road Traffic Injuries, The University of Queensland Brisbane QLD Australia; ^7^ Menzies Health Institute Queensland Griffith University Gold Coast QLD Australia; ^8^ Department of Physiotherapy Neurosurgery Spinal Clinic, Sir Charles Gairdner Hospital Perth WA Australia; ^9^ Faculty of Business Management and Social Sciences Hochschule Osnabrück, University of Applied Sciences Osnabrück Germany

## Abstract

**Background:**

This study describes a low‐cost and time‐efficient clinical sensory test (CST) battery and evaluates its concurrent validity as a screening tool to detect somatosensory dysfunction as determined using quantitative sensory testing (QST).

**Method:**

Three patient cohorts with carpal tunnel syndrome (CTS, *n* = 76), non‐specific neck and arm pain (NSNAP, *n* = 40) and lumbar radicular pain/radiculopathy (LR, *n* = 26) were included. The CST consisted of 13 tests, each corresponding to a QST parameter and evaluating a broad spectrum of sensory functions using thermal (coins, ice cube, hot test tube) and mechanical (cotton wool, von Frey hairs, tuning fork, toothpicks, thumb and eraser pressure) detection and pain thresholds testing both loss and gain of function. Agreement rate, statistical significance and strength of correlation (phi coefficient) between CST and QST parameters were calculated.

**Results:**

Several CST parameters (cold, warm and mechanical detection thresholds as well as cold and pressure pain thresholds) were significantly correlated with QST, with a majority demonstrating >60% agreement rates and moderate to relatively strong correlations. However, agreement varied among cohorts. Gain of function parameters showed stronger agreement in the CTS and LR cohorts, whereas loss of function parameters had better agreement in the NSNAP cohort. Other CST parameters (16 mN von Frey tests, vibration detection, heat and mechanical pain thresholds, wind‐up ratio) did not significantly correlate with QST.

**Conclusion:**

Some of the tests in the CST could help detect somatosensory dysfunction as determined with QST. Parts of the CST could therefore be used as a low‐cost screening tool in a clinical setting.

**Significance:**

Quantitative sensory testing, albeit considered the gold standard to evaluate somatosensory dysfunction, requires expensive equipment, specialized examiner training and substantial time commitment which challenges its use in a clinical setting. Our study describes a CST as a low‐cost and time‐efficient alternative. Some of the CST tools (cold, warm, mechanical detection thresholds; pressure pain thresholds) significantly correlated with the respective QST parameters, suggesting that they may be useful in a clinical setting to detect sensory dysfunction.

## 1 INTRODUCTION

Somatosensory dysfunction is common in various pain conditions, including neuropathic (Maier et al., 2010) and nociceptive pain (Moloney, Hall, & Doody, 2015; Tampin, Hall, & Briffa., 2012). Clinically, somatosensory dysfunction can present as loss or gain of sensory function. Loss of sensory function (hereby termed loss of function) manifests through decreased sensitivity, whereas gain of sensory function (hereby termed gain of function) includes hypersensitivity and/or spontaneous pain (Baron et al., 2017). Loss of function is an important feature of neuropathic pain and often associated with demylination or axon degeneration following nerve injury (Campbell, 2008; Schmid, Bland, Bhat, & Bennett, 2014). On the other hand, gain of function is indicative of neuronal hyperexcitability including lack of inhibition (Costigan, Scholz, & Woolf, 2009). The type of sensory dysfunction does not only provide important clues for diagnosis, but may also have prognostic implications. As an example, cold hyperalgesia and increased pain intensity is a predictor for poor recovery in patients with whiplash associated disorder (Sterling, Jull, Vicenzino, & Kenardy, 2003) and lateral epicondylalgia (Coombes, Bisset, & Vicenzino, 2015). Recent research work also suggests that sensory phenotyping could provide guidance in targeted management and possible stratified pharmacological management for patients with chronic pain (Forstenpointner, Otto, & Baron, 2018).

The current standard to evaluate the presence of a somatosensory dysfunction in clinical pain cohorts is quantitative sensory testing (QST). This method evaluates both loss and gain of function and comprehensively covers somatosensory sub‐modalities mediated by different primary afferents (C‐, Aδ‐, Aβ‐ fibres; Rolke et al., 2006). The standardized QST battery is widely acknowledged and frequently used in research settings as it allows the assessment of the whole spectrum of sensory nerve fibre dysfunction. However, from a clinical standpoint the equipment for QST is expensive and the procedure requires specialized training and is time consuming.

Because of costs and challenges of implementing QST in clinical practice, previous studies have strived to develop new tools to detect somatosensory dysfunction in a clinical setting. Although these studies provided important insights into the validity of low‐cost sensory testing tools, only a subset of sensory modalities were examined, thus not covering the full spectrum of somatosensory nerve function. Consequently, the objective of this study was to evaluate the concurrent validity of a comprehensive, low‐cost and time‐efficient clinical sensory test (CST) battery compared to the QST protocol for use as a screening tool to detect somatosensory dysfunction. We achieved this in a large population of patients with pain related to different clinical pain aetiologies and related mechanisms.

## 2 MATERIALS AND METHODS

### 2.1 Participants

A total of 142 patients were recruited from three patient cohorts; carpal tunnel syndrome (CTS), non‐specific neck and arm pain (NSNAP) and lumbar radicular pain with or without radiculopathy (LR). These cohorts were chosen to reflect a wide range of pain mechanisms including neuropathic and nociceptive pain, thus facilitating generalizability of results. For the CTS cohort, 76 patients were recruited from the Department of Neurology and Hand Surgery at the local University Hospital in Oxford, UK. All patients included in the CTS cohort met the electrodiagnostic (Bland, 2000) and clinical (Neurology QSSotAAo & Neurology QSSotAAo, 1993) criteria for CTS. Patients with peripheral neuropathy other than CTS (e.g. radial or ulnar neuropathy, cervical radiculopathy), other medical conditions that influence the cervical spine or upper extremity and a history of major surgery/trauma to the upper limb or neck were excluded. Patients with pregnancy or diabetes‐related CTS or with symptoms in the ipsilateral lateral upper arm were excluded as well. Forty‐four healthy subjects (proportionally matched for age and gender) were recruited in Oxford, UK and Gold Coast, Queensland, Australia to establish *z*‐scores for QST data in the CTS cohort. Part of the data from this cohort has previously been published (Baselgia, Bennett, Silbiger, & Schmid, 2017; Ridehalgh, Sandy‐Hindmarch, & Schmid, 2018; Schmid et al., 2014).

The NSNAP cohort consisted of 40 patients diagnosed with strictly unilateral NSNAP recruited from the Centre of Pain Medicine outpatient department of the Swiss Paraplegic Centre in Nottwil. Patients with specific causes for neck pain (e.g. abnormalities on bedside neurological examination or electrodiagnostic tests, specific MRI findings) were excluded. Other exclusion criteria included: diseases involving the nervous system (e.g. disorders of the central nervous system or diabetic neuropathy), previous upper limb or spinal surgery, major trauma affecting the upper limb and/or cervical region in the past two years, psychiatric or mental disorders. Thirty‐one healthy, gender and age matched subjects served as controls for the calculation of *z*‐scores in the NSNAP cohort (Tampin et al., 2012). This allowed matching of at least *n* = 8 healthy participants to each maximal pain area tested, a methodology that aligns with established methods documented by the German Research Network on Neuropathic Pain (DFNS) for data standardization (Blankenburg et al., 2010).

The LR cohort included 26 patients with unilateral radicular pain with or without motor and/or sensory loss of function recruited from the Neurosurgery Spinal Clinic at Sir Charles Gairdner Hospital, Perth, Western Australia. Patients were included if they had leg pain in the L5 or S1 dermatomal distribution and if the intensity of leg pain was higher than the intensity of back pain. Lumbar imaging was available for all patients (CT *n* = 5, MRI *n* = 21). Based on the grading system by Pfirrmann et al. (2004), 12 patients demonstrated nerve root compression of the clinically relevant nerve root, six patients demonstrated nerve root displacement and in eight patients the relevant nerve root was in contact with disc material. No patient was scheduled for surgery at the time of participation. The following exclusion criteria applied for the LR cohort: presence of diabetes, vascular disease; other neurological or psychiatric disease; symptom duration less then three months and an insufficient level of English to understand the instructions given during QST. Sixty‐two healthy participants, who participated in concurrent studies served as controls for the calculation of the *z*‐scores for the LR cohort. Thus, reference data were available of at least *n* = 8 healthy participants for each maximal pain area tested and for each age decade (30–39, 40–49, 50–65; Blankenburg et al., 2010).

The study was approved by the London Riverside national ethics committee (Ref Nr 10/H0706/35, for CTS cohort), The Ethics Committees Nordwest‐ und Zentralschweiz (EKNZ 2014‐243), the Ethics Committee of the Sir Charles Gairdner Osborne Park Health Care Group (HREC 2013‐096) and the Griffith University Human Research Ethics Committee (#2016/504). All participants gave written informed consent prior to attending a single appointment, during which demographic and clinical parameters (e.g. symptom severity and duration) were measured followed by the performance of both QST and CST. The CST was performed prior to QST. The investigators performing QST were blind to the outcome of CST in the NSNAP and LR cohorts.

### 2.2 Quantitative sensory testing

The QST was performed according to the standardized test protocol developed by the DFNS (Rolke et al., 2006). All investigators were trained by the DFNS. From the QST battery, we evaluated 10 parameters, which were classified into two categories: parameters for loss and gain of function. We did not include thermal sensory limen, paradoxical heat sensation and mechanical pain sensitivity because of the difficulty in mimicking these tests with low‐cost clinical tools. The QST battery took approximately 15–20 min for familiarization and 25–30 min to perform the actual test.

#### 2.2.1 Parameters for loss of function

Cold and warm detection thresholds (CDT, WDT) were examined with a thermotester (Somedic, Sweden). Three repetitions were performed and the average temperature was recorded (Rolke et al., 2006). Mechanical detection threshold (MDT) was evaluated using the geometric mean of five ascending and descending stimuli with von Frey filaments (Marstock, Germany). Vibration detection threshold (VDT) was recorded with a Rydel Seiffer tuning fork, which is graded on a scale from 0 to 8. Three measurements were performed to determine the mean VDT. The mechanical pain threshold (MPT) was tested with weighted pin prick stimulators (MRC). Five series of ascending and descending stimuli were performed and the geometric mean used for analysis. Since MPT reveals both loss and gain of function, this test was evaluated for both domains.

#### 2.2.2 Parameters for gain of function

Cold pain threshold (CPT) and heat pain thresholds (HPT) were evaluated with the thermotester (Somedic Sweden). Patients were tested three times and the mean was used for analysis. Pressure pain threshold (PPT) was tested with a manual algometer (Wagner Instruments) or digital algometer (Somedic). The mean of three repetitions was recorded. Mechanical pain threshold (MPT) was tested as described above. The wind‐up ratio (WUR) evaluates the effect of repeated stimuli, tested with a 256 mN pinprick stimulator. Patients were asked to rate the magnitude of pain evoked by the first stimulus and average of 10 repeated stimuli using a numerical pain rating scale (NRS) from 0 (no pain at all) to 100 (worst pain imaginable). The WUR was calculated by dividing the average NRS of 10 stimuli by the NRS of the first stimulus. Dynamic mechanical allodynia (ALL) was tested five times with a standardized brush (Somedic, Sweden), a cotton wisp and a Q‐tip. Subjects were asked to rate the pain intensity from 0 to 100 using an NRS as outlined above. The presence of allodynia was determined by any rating above 0.

All participants were familiarized with QST procedures on an unaffected control area (CTS: dorsum of ipsilateral hand, NSNAP/LR: dorsum of hand on asymptomatic side) before testing the affected pain area. For the CTS cohort, the affected pain area was standardized to the palmar side of the index finger reflecting an area innervated by the median nerve (Schmid et al., 2014). For the NSNAP and LR cohorts, we standardized the test site to the area of maximal pain indicated by each patient as not all patients have pain in a specific innervation territory (Haanpaa et al., 2011; Maier et al., 2010). The test areas of each cohort are listed in Table 1.

**Table 1:** Sensory test areas for each patient cohort. Data are provided as total number of patients (%)

	CTS cohort	NSNAP cohort	LR cohort
Palmar index finger	76 (100%)		
Upper trapezius		23 (57.5%)	
Cervical spine		9 (22.5%)	
Thoracic spine		6 (15.0%)	
Below spinae scapulae		2 (5.0%)	
Upper leg (L5)			2 (7.7%)
Upper leg (S1)			8 (30.8%)
Lower leg (L5)			7 (26.9%)
Lower leg (S1)			8 (30.8%)
Foot (S1)			1 (3.8%)

Abbreviations: CTS, carpal tunnel syndrome; LR, lumbar radicular pain/radiculopathy; NSNAP, non‐specific neck and arm pain.

### 2.3 CST Battery

**Figure 1:**
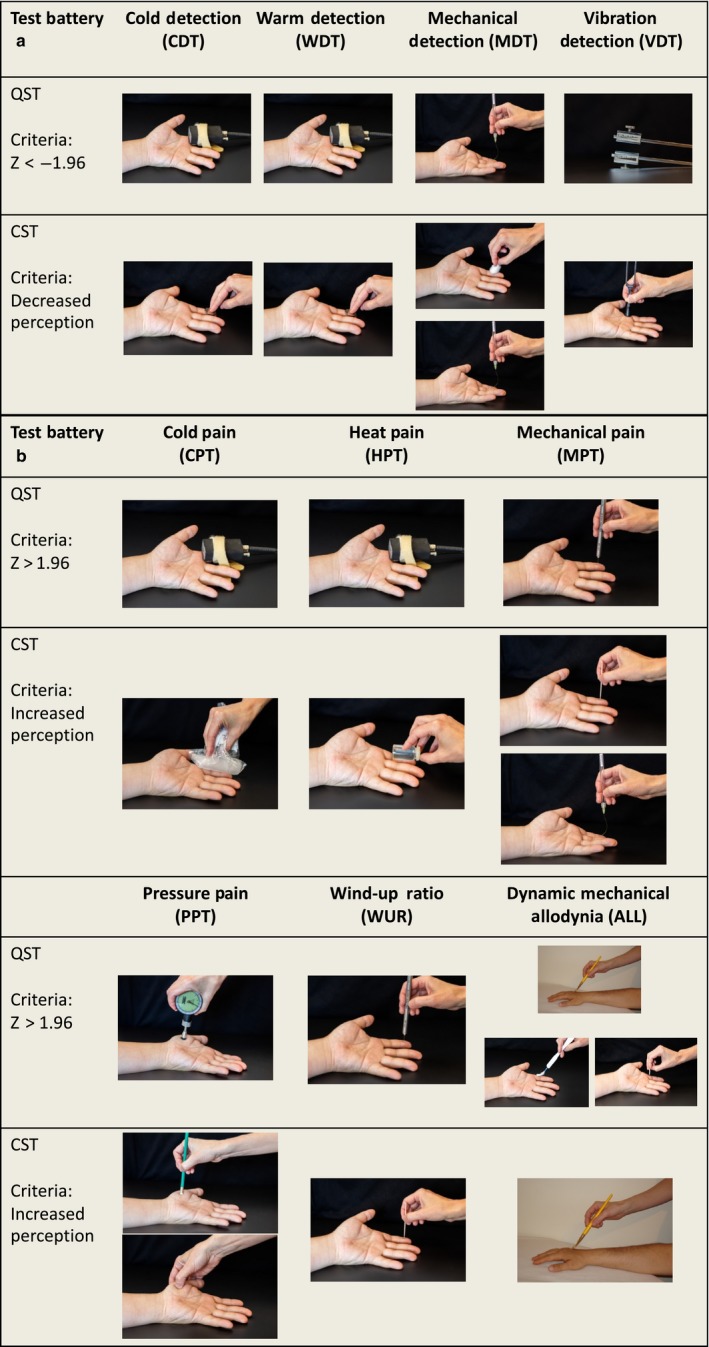
Parameters of the quantitative sensory testing (QST) and their respective clinical sensory test (CST) parameters. (a) Loss of function parameters, (b) Gain of function parameters

The CST battery consisted of 13 tests, each corresponding to a QST parameter while using readily available tools (Figure 1). Some QST parameters were represented by more than one CST test. The CST battery was based on the previously published standardized evaluation of pain (StEP) protocol (Scholz et al., 2009) as well as on previous work from our laboratory (Ridehalgh et al., 2018). Participants were tested on an unaffected, symptom‐free control area first (CTS: ipsilateral lateral upper arm, NSNAP/LR: contralateral mirror site) followed by the affected area as outlined in the QST methodology above (CTS: index finger, NSNAP/LR: maximal pain area). Each test was performed once at both the test and control area during the examination. The order of the CST followed the same sequence as test parameters of the QST protocol. The testing took about 10–15 min.

#### 2.3.1 Parameters for loss of function

Cold/warm detection thresholds (CDT^CST^/WDT^CST^): This set of tests was performed with metal coins (50 UK pence, 50 AUS cents) as previously described (Ridehalgh et al., 2018). For CDT^CST^, a coin which was held at room temperature was used while WDT^CST^ was tested with a coin placed in the pocket of the investigator for 30 min.

Mechanical detection threshold (MDT^CST^): MDT^CST^ consisted of two tests, sensitivity to light stroke with a cotton wool and sensitivity to a von Frey filament weighing 16 mN (Scholz et al., 2009).

Vibration detection threshold (VDT^CST^): The VDT^CST^ was evaluated with a tuning fork of 128 Hz frequency. The amplitude of the tuning fork was standardized by releasing the metal fork from a fully approximated position.

Mechanical pain threshold (MPT^CST^): The MPT^CST^ was evaluated with two modalities; a toothpick and a von Frey filament weighing 256 mN (Scholz et al., 2009).

#### 2.3.2 Parameters for gain of function

Cold/heat pain thresholds (CPT^CST^/HPT^CST^): For CPT^CST^, ice cubes were placed in a plastic bag on the patients’ skin for 10 s. For HPT^CST^, a glass vial filled with hot tap water (40°C) was placed over the skin for 10 s.

Mechanical pain threshold (MPT^CST^): The MPT^CST^ for gain of function was evaluated with two modalities: a toothpick and a von Frey filament (256mN) as described for MPT^CST^ loss of function above.

Pressure pain threshold (PPT^CST^): The PPT^CST^ was evaluated with two modalities; with an eraser mounted on a pencil (7 mm diameter) and with the examiner's thumb. The eraser/thumb was placed over the testing area and pressure was applied for 10 s (Scholz et al., 2009). The pressure was sufficient to indent the soft tissues and lead to skin blanching.

Wind‐up Ratio (WUR^CST^): The WUR^CST^ was established with a toothpick by applying a single stimulus followed by a train of 10 stimuli with the participants rating the ensuing pain on a NRS from 0 (no pain at all) to 100 (worst pain imaginable). As per QST protocol, the wind‐up ratio was calculated as the ratio of the two pain ratings.

Dynamic mechanical allodynia (ALL^CST^):

Dynamic mechanical allodynia was assessed with a brush (Somedic, Sweden) and patients were asked whether or not this produced a painful response.

#### 2.3.3 CST interpretation

For both loss and gain of function CST parameters, patients were asked whether the stimulus applied at the affected site was perceived as increased, decreased or the same intensity compared to the symptom‐free control area. In the loss of function parameters (detection thresholds and MPT) a perception of decreased sensation was interpreted as loss of function. In the gain of function parameters (pain thresholds and WUR), a perception of increased sensation was considered to reflect gain of function. For the gain of function parameters, the intensity of pain was also recorded on a NRS scale ranging from 0 (no pain at all) to 10 (worst pain imaginable). Allodynia was rated as present or absent. The NRS score was analysed separately to investigate if the intensity of pain alone could help identify patients with gain of function.

### 2.4 Data transformation

Quantitative sensory testing parameters except for CPT, HPT and VDT were log‐transformed to achieve normal distribution. The *z*‐scores were calculated using the following formula: *z*‐score = (Value_patient_ – Mean_control_/Standard deviation_control_) (Rolke et al., 2006). *Z*‐scores below zero indicate loss of function, whereas *z*‐scores above zero indicate gain of function. In accordance with traditional practice, we defined a *z*‐score over 1.96 (gain of function) or below −1.96 (loss of function) as a clinically relevant sensory dysfunction (Rolke et al., 2006). We have previously shown that patients with CTS, NSNAP and radiculopathies have clearly abnormal QST *z*‐scores compared to healthy participants while *z*‐scores mostly remain within the traditional 1.96 standard deviation cut‐offs (Schmid et al., 2014; Tampin et al., 2012; Tampin, Vollert, & Schmid, 2018). We have therefore performed a supplementary sensitivity analysis using a ±1.0 standard deviation cut‐off to determine sensory dysfunction in *z*‐scores. This cut‐off presumably includes more false positives, whereas the traditional cut‐off has the risk of classifying more false‐negatives. Both QST and CST data were transformed into binary data (positive or negative) to facilitate the calculation of agreement rate as well as the significance and strength of correlation. For QST, a *z*‐score smaller than −1.96 (for sensitivity analysis <−1.0) in loss of function tests and >1.96 (for sensitivity analysis >1.0) in gain of function tests was defined as a sensory dysfunction (positive). For CST, a perceived decreased response on loss of function tests and increased response on gain of function tests compared to the control area was defined as a sensory dysfunction (positive).

### 2.5 Statistical analysis

Data were analyzed for the mixed cohort as well as each cohort separately using SPSS 25 (IBM). Two‐by‐two contingency tables were used to calculate the agreement rate and correlations between QST and CST parameters. Fisher's exact test was used to determine significant correlations between the outcomes of QST and CST in all parameters. In order to assess the strength of correlations between the corresponding QST and CST variables, Phi correlation coefficients were calculated. A Phi coefficient between 0 and 0.1 indicates negligible correlation, 0.1–0.2 weak, 0.2–0.4 moderate, 0.4–0.6 relatively strong association, 0.6–0.8 strong association and >0.8 very strong association between two binary variables (Rea & Parker, 2014).

The NRS score collected from the gain of function parameters was analysed separately using receiver operating characteristic (ROC) curves to assess if the NRS scores are indicative of gain of function as determined with QST. For those parameters with an area under curve (AUC) exceeding 0.7 indicating acceptable discriminative power (Mandrekar, 2010), sensitivity and specificity were calculated.

## 3 RESULTS

The demographic and clinical details of the patient cohorts are described in Table 2 and of the healthy controls in Table S1. The number and relative frequency of patients classified as loss or gain of function in QST and CST are summarized in Table S2. The agreement rate, outcome of Fisher's exact test and the Phi coefficient of the analyses using the traditional 1.96 QST *z*‐score cut‐off are summarized in Table 3A‐D.

**Table 2:** Demographic and clinical characteristics of the patient cohorts. Data are provided as mean and standard deviation unless indicated otherwise

	CTS	NSNAP	LR
Number of participants	76	40	26
Age in years	61.4 (12.6)	46 (12.0)	46.5 (9.9)
Female *n* (%)	51 (67.1%)	28 (70.0%)	10 (38.5%)
Symptom duration in months	62.5 (87.7)	72.35 (44.6)	19.3 (23.5)
Current pain intensity (NRS 0–10)	1.6 (2.2)	3.3 (2.4)	3.0 (1.8)
PainDETECT	11.7 (4.2)	13.9 (6.3)	15.2 (7.3)
Boston symptom questionnaire	2.7 (0.7)	N/A	N/A
Boston function questionnaire	2.2 (0.8)	N/A	N/A
Neck disability index	N/A	26.0 (7.3)	N/A
Oswestry disability index	N/A	N/A	32.1 (15.6)

Note

PainDETECT questionnaire to determine the presence of neuropathic pain (≦12 unlikely neuropathic component, ≧19 likely neuropathic component); Boston symptom/function questionnaire for patients with CTS (1 no symptom or function deficit, 5 severe pain or function deficit); Neck disability index for patients with NSNAP (0–4 no disability; 5–14 mild disability; 15–24 moderate disability; 25–34 severe disability; >34 complete disability); Oswestry disability index for the LR cohort (0%–20% minimal disability; 21%–40% moderate disability; 41%–60% severe disability; 61%–80% crippled; 81%–100% bed bound or exaggerating symptoms).

Abbreviations: CTS, carpal tunnel syndrome; LR, lumbar radicular pain/radiculopathy; NRS, numerical pain rating scale (0 no pain at all, 10 worst pain imaginable); NSNAP, non‐specific neck and arm pain.

**Table 3:** Concurrent validity parameters of the clinical sensory testing compared to quantitative sensory testing in the carpal tunnel syndrome CTS (A), non‐specific neck arm pain NSNAP (B), lumbar radicular pain/radiculopathy LR (C) and mixed cohort (D). Criteria for sensory dysfunction in QST: Z > 1.96 (gain of function) or Z < −1.96 (loss of function). Criteria for sensory dysfunction in CST: patient reported increased (gain of function) or decreased (loss of function) response compared to control area

Parameters A‐(CTS)	Agreement %	Fisher's exact test	Phi Coefficient
Loss of function			
CDT^CST^	46.1%	0.547	0.094
WDT^CST^	53.9%	0.195	0.162
MDT^CST^Cotton	55.3%	0.364	0.110
MDT^CST^VF16	60.5%	0.107	0.191
VDT^CST^	47.4%	0.817	−0.039
MPT^CST^(LoF)	65.8%	N/A	N/A
Gain of function			
CPT^CST^	69.7%	**0.01**	**0.311**
HPT^CST^	62.7%	0.546	−0.144
PPT^CST^Eraser	84.2%	0.438	0.083
PPT^CST^Thumb	86.8%	0.365	0.111
MPT^CST^	52.6%	0.725	−0.050
MPT^CST^VF256	76.3%	0.354	0.138
WUR^CST^	70.3%	1.000	−0.067

Parameters B‐(NSNAP)	Agreement %	Fisher's exact test	Phi Coefficient
Loss of function			
CDT^CST^	77.5%	**0.014**	**0.420**
WDT^CST^	80.0%	0.448	0.119
MDT^CST^Cotton	77.5%	0.498	0.095
MDT^CST^VF16	57.5%	0.539	−0.209
VDT^CST^	72.5%	0.056	0.330
MPT^CST^(LoF)	85.0%	N/A	N/A
Gain of function			
CPT^CST^	45.0%	0.752	−0.098
HPT^CST^	45.0%	0.512	−0.144
PPT^CST^Eraser	60.0%	0.095	0.306
PPT^CST^Thumb	57.5%	0.680	0.112
MPT^CST^	60.0%	N/A	N/A
MPT^CST^VF256	50.0%	N/A	N/A
WUR^CST^	47.5%	N/A	N/A

Parameters C‐(LR)	Agreement %	Fisher's exact test	Phi Coefficient
Loss of function			
CDT^CST^	61.5%	0.365	0.225
WDT^CST^	69.2%	0.095	0.358
MDT^CST^Cotton	69.2%	0.109	0.350
MDT^CST^VF16	53.8%	0.683	0.137
VDT^CST^	50.0%	0.614	−0.149
MPT^CST^(LoF)	53.8%	0.683	0.116
Gain of function			
CPT^CST^	60.0%	1.000	0.016
HPT^CST^	69.2%	0.529	0.120
PPT^CST^Eraser	70.8%	0.552	0.205
PPT^CST^Thumb	72.0%	0.156	0.336
MPT^CST^	84.6%	N/A	N/A
MPT^CST^VF256	96.2%	N/A	N/A
WUR^CST^	76.5%	1.000	−0.116

Parameters D‐(Mixed)	Agreement %	Fisher's exact test	Phi Coefficient
Loss of function			
CDT^CST^	57.7%	**0.003**	**0.249**
WDT^CST^	64.1%	**0.016**	**0.214**
MDT^CST^Cotton	64.1%	**0.01**	**0.231**
MDT^CST^VF16	58.5%	0.149	0.129
VDT^CST^	54.9%	0.381	0.086
MPT^CST^(LoF)	69.0%	0.112	0.157
Gain of function			
CPT^CST^	61.0%	0.165	0.132
HPT^CST^	58.9%	0.360	−0.083
PPT^CST^Eraser	75.0%	**0.017**	**0.222**
PPT^CST^Thumb	75.9%	**0.016**	**0.223**
MPT^CST^	60.6%	1.000	−0.003
MPT^CST^VF256	72.5%	0.429	0.064
WUR^CST^	64.1%	0.434	−0.103

Note

Parameters with significance in Fisher's exact test and greater than negligible correlation are marked in bold.

Abbreviations: CDT, cold detection threshold; CPT, cold pain threshold; CST, clinical sensory testing; HPT, heat pain threshold; LoF/GoF, loss/gain of function; MDT, mechanical detection threshold; MPT, mechanical pain threshold; PPT, pressure pain threshold; QST, quantitative sensory testing; VDT, vibrational detection threshold; VF16, von Frey hair weighing 16 mN; VF256, von Frey hair weighing 256 mN; WDT, warm detection threshold; WUR, wind‐up ratio.

### 3.1 Agreement of CST with QST

The overall agreement rate in single and mixed cohorts ranged from 45.0% to 96.2%. Each cohort had distinct parameters that showed a high agreement rate of >80%. Loss of function parameters (WDT^CST^ and MPT^CST^) conformed best in the NSNAP cohort (80%–85%; Table 3B). Gain of function parameters, namely PPT^CST^ Thumb, PPT^CST^ Eraser in the CTS cohort (84%–87%) and MPT^CST^, MPT^CST^VF256 in the LR cohort (85%–96%) showed strong agreement (Table 3A and C). The mixed cohort yielded agreement rates for loss of function parameters between 55% and 69% and for gain of function parameters between 59% and 76% (Table 3D).

### 3.2 Correlation of CST with QST

Six parameters (CDT^CST^, WDT^CST^, MDT^CST^‐cotton wool, CPT^CST^, PPT^CST^Eraser and PPT^CST^Thumb) showed a significant correlation ranging from moderate (0.214) to relatively strong (0.420). Loss of function parameters of CDT^CST^ showed a relatively strong correlation with QST in the NSNAP cohort (0.420) and moderate correlation in the mixed cohort (0.249) and WDT^CST^ and MDT^CST^‐cotton wool were moderately correlated with QST in the mixed cohort (0.214 and 0.231, respectively). For the CTS and LR cohort, no loss of function parameters correlated significantly with QST measures.

The gain of function parameter CPT^CST^ showed a moderate correlation with its QST counterpart in the CTS cohort (0.311). In the NSNAP and LR cohort, none of the gain of function CST parameters correlated significantly with QST. For the mixed cohort, PPT^CST^Eraser and PPT^CST^Thumb demonstrated moderate correlations (0.222 and 0.223, respectively; Table 3D).

For several parameters, correlations could not be calculated because of uneven patient distribution (Table 3A‐C): loss of function MPT^CST^ in CTS and NSNAP cohort, gain of function MPT^CST^VF256, WUR^CST^ in the NSNAP cohort and MPT^CST^ and MPT^CST^VF256) in the LR cohort. For example, the MPT^CST^VF256 in the LR cohort revealed 96.2% agreement with the corresponding QST parameter, however none of the patients in the LR cohort was classified as positive in the QST. It was therefore not possible to calculate Fisher's exact tests and the strength of correlations. We did not conduct analyses on dynamic mechanical allodynia (ALL) as no patient had allodynia as determined with QST and only three patients with the CST (one from the CTS cohort, two from the NSNAP cohort).

### 3.3 NRS scale analysis

**Figure 2:**
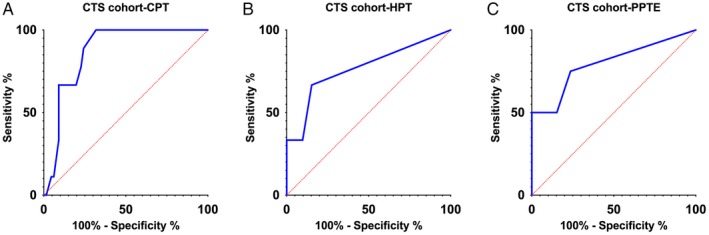
Receiver operating curve of (a) cold pain threshold (CPT), (b) heat pain threshold (HPT) and (c) pressure pain threshold assessed with an eraser (PPTE) in the carpal tunnel syndrome cohort. Data generated using the 1.96 *SD* cut‐off for quantitative sensory testing *z*‐scores

The ROC analysis revealed three parameters with acceptable discriminative power to identify patients with gain of function. The NRS score of CPT^CST^, HPT^CST^ and PPT^CST^Eraser in the CTS cohort yielded AUCs of 0.869, 0.766 and 0.797, respectively. The ROC analysis indicated that an NRS value >0.5 in the CPT^CST^ has 100% sensitivity and 68.2% specificity to detect cold hyperalgesia as determined with QST in patients with CTS. The ROC curve of HPT^CST^ and PPT^CST^Eraser suggested that an NRS value >0.5 has 66.7% and 75% sensitivity and 84.7% and 76.4% specificity to detect heat and pressure hyperalgesia, respectively (Figure 2a‐c).

### 3.4 Supplementary sensitivity analysis using 1 standard deviation cut‐off

The supplementary sensitivity analysis using a more lenient cut‐off criteria of 1 standard deviation for the QST *z*‐scores revealed comparable results for agreement rates and correlations (Table S3A‐D) as the more conservative 1.96 standard deviation cut‐off. The ROC analysis using the criteria of 1 standard deviation revealed that only PPT^CST^Thumb in the NSNAP cohort had acceptable discriminative power to identify patients with pressure hyperalgesia (Figure S1). However, the overall pattern of AUC was comparable with the results calculated with the 1.96 standard deviation cut‐off (Table S4).

## 4 DISCUSSION

Our study investigated the concurrent validity of a comprehensive, low‐cost and time‐efficient CST battery compared to a standardized QST in a cohort of patients with mixed diagnoses. Certain CST parameters (CDT^CST^, WDT^CST^, MDT^CST^‐Cotton wool, CPT^CST^, PPT^CST^Eraser and PPT^CST^Thumb) were significantly correlated with the outcome of QST, yielding moderate to relatively strong correlations and mostly over 60% agreement rate. Other CST parameters have however not shown significant correlations with the outcome of QST (loss of function: MDT^CST^VF16, VDT^CST^, MPT^CST^, gain of function: HPT^CST^, MPT^CST^, MPT^CST^VF256, WUR^CST^) which may query their value in clinical practice for these patient cohorts. The agreement and correlation of CST parameters with QST varies substantially not only between different parameters, but also among different patient cohorts.

A closer inspection of the parameters revealed that comparative tests analysing loss of function had stronger agreement in the NSNAP cohort, whereas comparative tests analysing gain of function parameters were superior in the CTS and LR cohorts. These findings were largely driven by the high proportion of negative findings in the respective sensory parameters (e.g. mostly negative findings in loss of function parameters in the NSNAP cohorts and mostly negative findings in gain of function parameters in the CTS and LR cohorts). This pattern reflects previously published data, which suggest that loss of function is the predominant phenotype in patients with actual nerve lesions resulting in neuropathic pain such as in our CTS (Schmid et al., 2014) and lumbar radiculopathy cohorts (Freynhagen et al., 2008; Tampin, Slater, & Lind, 2017; Tschugg et al., 2016) whereas patients with predominant nociceptive pain such as our NSNAP cohort are mostly characterised by gain of function (Moloney, Hall, & Doody, 2013).

While previous research has evaluated low‐cost alternatives of QST for clinical use, most studies were aimed at identifying a specific condition, for instance radicular pain (Scholz et al., 2009) or small fibre degeneration (Haussleiter et al., 2008; Ridehalgh et al., 2018). We are aware of only three studies which made a direct comparison of simple CSTs with standardized QST (Buliteanu et al., 2018; Haussleiter et al., 2008; Leffler & Hansson, 2008). One study identified a 78% agreement rate of QST with the NeuroQuick, which uses wind chill (a combination of air temperature and wind speed) to determine cold detection thresholds (Haussleiter et al., 2008). However, no other sensory parameters were evaluated. Buliteanu et al. (2018) compared clinical bedside tests with QST in patients with chronic neuropathic pain of different aetiologies. At the time of writing this manuscript, only the abstract was available, reporting a high correlation for MPT, temporal summation and CPT. It remains unclear, whether detection thresholds (for evaluating loss of function) were included in their study. Leffler and Hansson (2008 ) described a bedside examination battery evaluating mechanical allodynia and cold/warm thresholds in a small cohort of patients (*n* = 32) with traumatic nerve injury. Based on the reported agreement rates ranging from 48% to 58%, the authors concluded that the outcome of bedside sensory testing and QST often differs. Despite including patients with presumably more subtle sensory changes due to non‐traumatic aetiologies, our agreement rates are higher, especially in the NSNAP and LR cohort. In contrast to Leffler and Hansson (2008), we discriminated between loss and gain of function, which have distinct underlying mechanisms with potentially different clinical relevance in relation to diagnosis and prognosis (Arendt‐Nielsen et al., 2018; Coombes et al., 2015; Sterling et al., 2003).

While QST is commonly accepted as the standardized tool to detect sensory dysfunction, it remains a psychophysical tool that is dependent on internal as well as external influences and may thus not represent a true gold standard. The traditional cut‐off point for abnormality of QST *z*‐scores of 1.96 standard deviations (Rolke et al., 2006) has led to the skewedness of several parameters (e.g. no patients classified as having an abnormality in QST). Having previously found that patients with CTS, NSNAP and LR have clearly abnormal QST *z*‐scores compared to gender and age matched controls despite often not exceeding the traditional two standard deviation threshold (Schmid et al., 2014; Tampin et al., 2012), we also provided a sensitivity analysis using a 1 *SD* cut‐off. Of note, using the more lenient cut‐off (1 *SD*) revealed comparable agreement rates and correlations between CST and QST as the conservative 1.96 *SD* cut‐off (Table S4A‐D), lending further support to the stability of our results. This is likely due to the small number of patients falling within the 1–1.96 standard deviation range.

In our dataset, the CST classified more patients as having a sensory dysfunction than the QST in most parameters. A similar pattern was also reported by Leffler and Hansson (2008), who used a comparable approach in their bedside examination. This may suggest a greater number of false positive test outcomes, potentially related to the higher dependency on subjectiveness in the CST than the QST battery. On the other hand, it could be argued that the CST may detect more subtle sensory changes as the CST is not a continuous variable with a defined cut‐off, but is compared by the patients to a control area. As such, more patients may be classified as having abnormal sensory profiles with the CST battery as any change, however subtle, will be rated as abnormal. Whereas the issue of false positives needs to be considered, the detection of subtle sensory dysfunction is important as it may identifiy patients with subclinical signs of a neuropathy such as reported in pseudoradicular low back pain (Freynhagen et al., 2008) and it may also assist in targeting management more specifically (Baron, 2006).

In addition to exploring changes in perception to the stimulus (increased, decreased, the same intensity), we speculated that the pain intensity on an NRS can possibly be used to identify patients with hyperalgesia. However, this was only the case for measurements of CPT^CST^, HPT^CST^ and PPT^CST^Eraser in the CTS cohort. Nevertheless, the identification of thermal and pressure hyperalgesia is of clinical revelance, as cold hyperalgesia may be an indicator for poor prognosis in whiplash associated disorders (Sterling et al., 2003) and lateral epicondylalgia (Coombes et al., 2015), whereas a lower PPT may predict poor recovery after total knee replacement surgery (Arendt‐Nielsen et al., 2018).

### 4.1 Limitations and future directions

This study is an important first step in the development of a low‐cost screening tool to detect somatosensory dysfunction in a clinical setting. However, it would currently not substitute QST, whose quantitative characteristics renders it useful not only as a screening, but also as an outcome measure tool. The identified agreement rates and correlations suggest that parts of the CST are valuable as a clinical screening tool. Future studies are however required to determine whether CST can reliably be used to quantify the identified sensory dysfunctions, so that it can eventually serve as an outcome measure tool.

There are some methodological issues to be considered. Although we included three cohorts of patients representing different pain mechanisms and aetiologies, the sample sizes of these cohorts differed. Although agreement rates were comparable between cohorts, the relatively low number of patients in the NSNAP and LR cohorts may have prevented the identification of significant CST parameters in the ROC analysis. Also, we used the contralateral side as a control side for the CST in all cohorts except for CTS patients, where we used the ipsilateral lateral upper arm. Detection threholds are generally higher over the lateral upper arm than the distal hand (Heldestad Lillieskold & Nordh, 2018). This may have accounted for the somewhat lower agreement rates especially for loss of function tests in the CTS cohort. Nevertheless, the absence of a normal contralateral area as a control site represents a common clinical challenge, as up to 87% of patients with CTS have bilateral symptoms (Padua, Padua, Nazzaro, & Tonali, 1998). In addition, the area tested by the thermode was smaller in CTS patients compared to the NSNAP and LR cohorts, therefore covering a smaller area of receptive fields. The use of *z*‐scores is likely to correct for these differences, as they are based on healthy control data collected in comparably sized areas. We cannot exclude that the smaller areas examined during thermal testing in the CTS cohort may have contributed in part to the lower agreement rates especially for the loss of function tests.

A potential limitation in our study is that the investigator performing the QST was blinded to the CST outcome in the LR and NSNAP but not the CTS cohort. However, the QST *z*‐scores used for analyses remained unknown to the investigator, as they required additional QST data from healthy participants. Thus at the time of performing QST, the investigator was unaware whether or not participants would be classified as having abnormal *z*‐scores.

## 5 CONCLUSION

Our study reveals that a subset of tools in the low‐cost CST battery may be valid as a low‐cost screening tool to identify somatosensory dysfunction in a clincial setting, allowing for wider clinical use and more time‐efficient somatosensory phenotyping. However, not all CST tools proved to be useful and there is substantial variability between different patient cohorts, suggesting that certain CST parameters may be most relevant in specific cohorts.

## CONFLICT OF INTEREST

All authors have no conflict of interest in the subject matter or materials discussed in this manuscript.

## AUTHOR CONTRIBUTIONS

BT and AS conceptualized the experiment and acquired funding. GL, HS and KB provided input to the design of the study. KB, AS, BT, LH and SF collected data. GC, AS, BT and CC were involved in data analysis. GC and AS drafted the first version of the manuscript. All authors provided input to the final version of the manuscript.
