# Supplementary figures and images for: Concurrent validity of a low‐cost and time‐efficient clinical sensory test battery to evaluate somatosensory dysfunction
Source: Eur J Pain. 2019 Aug 28;23(10):1826–38. doi: 10.1002/ejp.1456 (PMC6852113; doi:10.1002/ejp.1456)

(1SD cut-off)  
NSNAP cohort-PPTT

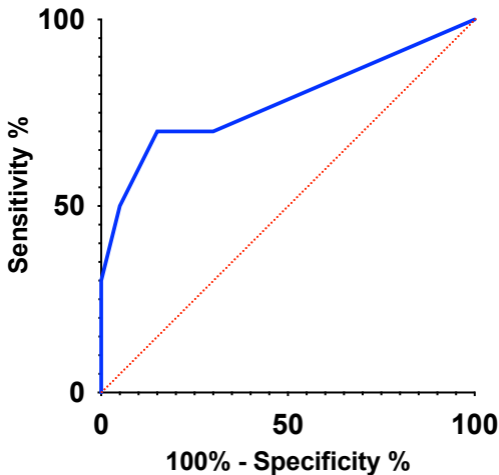

Supplement: Supplementary file 1 [file EJP-23-1826-s001.pdf]
