# Supplementary material for: Concurrent validity of a low‐cost and time‐efficient clinical sensory test battery to evaluate somatosensory dysfunction
Source: Eur J Pain. 2019 Aug 28;23(10):1826–38. doi: 10.1002/ejp.1456 (PMC6852113; doi:10.1002/ejp.1456)
Supplement: Supplementary file 2 [file EJP-23-1826-s002.docx]

Table S1: Age and gender of healthy participants proportionally matched to each patient cohort

|  | HC-CTS | HC-NSNAP | HC-LR |
| --- | --- | --- | --- |
| Number of participants | 44 | 31 | 62 |
| Age (years) (SD) | 57.1 (11.9) | 45.6 (12.5) | 43.7 (9.6) |
| Female n (%) | 29 (65.9%) | 15 (48%) | 32 (51.6%) |

HC: Healthy control; CTS: carpal tunnel syndrome; NSNAP: nonspecific neck and arm pain; LR: lumbar radicular pain/radiculopathy.

Table S2. The number (n) and relative frequency of patients with loss/gain of function in quantitative sensory testing (QST) and clinical sensory testing (CST) parameters. Criteria for sensory dysfunction in QST: Z >1.96 (gain of function) or Z<-1.96 (loss of function). Criteria for sensory dysfunction in CST: patient reported increased (gain of function) or decreased (loss of function) response compared to control area.

|  | QST | | CST | |
| --- | --- | --- | --- | --- |
| Parameters | n/Total | Relative  Frequency | n/Total | Relative frequency |
| Loss of function |  |  |  |  |
| CDT^CST^ | 45/142 | 31.7% | 85/142 | 59.9% |
| WDT^CST^ | 34/142 | 23.9% | 57/142 | 40.1% |
| MDT^CST^Cotton | 46/142 | 32.4% | 57/142 | 40.1% |
| MDT^CST^VF16 | 46/142 | 32.4% | 61/142 | 43.0% |
| VDT^CST^ | 51/142 | 35.9% | 67/142 | 47.2% |
| MPT^CST^(LoF) | 8/142 | 5.6% | 46/142 | 32.4% |
| Gain of function |  |  |  |  |
| CPT^CST^ | 36/141 | 25.5% | 55/141 | 39% |
| HPT^CST^ | 26/141 | 18.4% | 44/141 | 31.2% |
| PPT^CST^Eraser | 18/140 | 12.9% | 35/140 | 25.0% |
| PPT^CST^Thumb | 19/141 | 13.5% | 33/141 | 23.4% |
| MPT^CST^ | 8/142 | 5.6% | 54/142 | 38.0% |
| MPT^CST^VF256 | 8/142 | 5.6% | 37/142 | 26.1% |
| WUR^CST^ | 8/131 | 6.1% | 41/131 | 31.3% |

QST: quantitative sensory testing; CST: clinical sensory testing; CDT: cold detection threshold; WDT: warm detection threshold; MDT: mechanical detection threshold; VDT: vibrational detection threshold; CPT: cold pain threshold; HPT: heat pain threshold; PPT: pressure pain threshold; MPT: mechanical pain threshold; WUR: wind-up ratio; LoF: loss of function; VF16: von Frey hair weighing 16mN; VF256: von Frey hair weighing 256mN

Table S3. Concurrent validity of the clinical sensory test battery compared to quantitative sensory testing in the carpal tunnel syndrome (CTS, A), non-specific neck arm pain (NSNAP, B), lumbar radicular pain/radiculopathy (LR, C) and mixed cohort (D). Criteria for sensory dysfunction in QST: Z >1 (gain of function) or Z<-1 (loss of function). Criteria for sensory dysfunction in CST: patient reported increased (gain of function) or decreased (loss of function) response compared to control area.

| Parameters  A-(CTS) | Agreement % | Fisher’s exact test | | Phi  Coefficient |
| --- | --- | --- | --- | --- |
| Loss of function |  | |  |  |
| CDT^CST^ | 64.5% | | 1.000 | -0.039 |
| WDT^CST^ | 57.9% | | 0.167 | 0.170 |
| MDT^CST^Cotton | 56.6% | | 0.348 | 0.129 |
| MDT^CST^VF16 | 56.6% | | 0.155 | 0.177 |
| VDT^CST^ | 52.6% | | 0.817 | 0.039 |
| MPT^CST^(LoF) | 63.2% | | 0.387 | 0.104 |
| Gain of function |  | |  |  |
| CPT^CST^ | 67.1% | | **0.037** | **0.269** |
| HPT^CST^ | 60% | | 0.742 | -0.053 |
| PPT^CST^Eraser | 69.7% | | 0.704 | 0.045 |
| PPT^CST^Thumb | 69.7% | | 1.000 | 0.000 |
| MPT^CST^ | 53.9% | | 1.000 | 0.013 |
| MPT^CST^VF256 | 72.4% | | 0.454 | 0.108 |
| WUR^CST^ | 68.9% | | 0.725 | 0.064 |

| Parameters  B-(NSNAP) | Agreement % | Fisher’s exact test | Phi  Coefficient |
| --- | --- | --- | --- |
| Loss of function |  |  |  |
| CDT^CST^ | 65.0% | 0.273 | 0.217 |
| WDT^CST^ | 62.5% | 1.000 | -0.01 |
| MDT^CST^Cotton | 57.5% | 1.000 | 0.000 |
| MDT^CST^VF16 | 52.5% | 1.000 | -0.027 |
| VDT^CST^ | 57.5% | 0.286 | 0.219 |
| MPT^CST^(LoF) | 82.5% | 1.000 | -0.067 |
| Gain of function |  |  |  |
| CPT^CST^ | 47.5% | 0.748 | -0.068 |
| HPT^CST^ | 45.0% | 0.538 | -0.109 |
| PPT^CST^Eraser | 62.5% | 0.205 | 0.250 |
| PPT^CST^Thumb | 65.0% | 0.111 | 0.302 |
| MPT^CST^ | 57.5% | 1.000 | 0.000 |
| MPT^CST^VF256 | 52.5% | 1.000 | 0.076 |
| WUR^CST^ | 47.5% | N/A | N/A |

| Parameters  C-(LR) | Agreement % | Fisher’s  exact test | Phi  Coefficient |
| --- | --- | --- | --- |
| Loss of function |  |  |  |
| CDT^CST^ | 65.4% | 0.218 | 0.299 |
| WDT^CST^ | 73.1% | **0.043** | **0.443** |
| MDT^CST^Cotton | 61.5% | 0.053 | 0.433 |
| MDT^CST^VF16 | 61.5% | 0.644 | 0.130 |
| VDT^CST^ | 53.8% | 1.000 | 0.007 |
| MPT^CST^(LoF) | 69.2% | 0.105 | 0.378 |
| Gain of function |  |  |  |
| CPT^CST^ | 60.0% | 0.653 | 0.115 |
| HPT^CST^ | 61.5% | 0.203 | 0.312 |
| PPT^CST^Eraser | 54.2% | 1.000 | 0.037 |
| PPT^CST^Thumb | 56.0% | 0.673 | 0.114 |
| MPT^CST^ | 84.6% | N/A | N/A |
| MPT^CST^VF256 | 96.2% | N/A | N/A |
| WUR^CST^ | 70.6% | 0.515 | 0.304 |

| Parameters  D-(Mixed) | Agreement % | Fisher’s  exact test | Phi  Coefficient |
| --- | --- | --- | --- |
| Loss of function |  |  |  |
| CDT^CST^ | 64.8% | **0.002** | **0.276** |
| WDT^CST^ | 62% | **0.015** | **0.213** |
| MDT^CST^Cotton | 57.7% | **0.038** | **0.188** |
| MDT^CST^VF16 | 56.3% | 0.088 | 0.150 |
| VDT^CST^ | 54.2% | 0.401 | 0.084 |
| MPT^CST^(LoF) | 69.7% | **0.003** | **0.26** |
| Gain of function |  |  |  |
| CPT^CST^ | 60.3% | 0.054 | 0.176 |
| HPT^CST^ | 56% | 0.696 | -0.037 |
| PPT^CST^Eraser | 65% | **0.041** | **0.189** |
| PPT^CST^Thumb | 66% | **0.022** | **0.211** |
| MPT^CST^ | 60.6% | 0.607 | 0.05 |
| MPT^CST^VF256 | 71.1% | 0.248 | 0.111 |
| WUR^CST^ | 62.6% | 1.000 | -0.012 |

Parameters with significance in Fisher’s exact test and greater than negligible correlation are marked in bold.QST: quantitative sensory testing; CST: clinical sensory testing; CDT: cold detection threshold; WDT: warm detection threshold; MDT: mechanical detection threshold; VDT: vibrational detection threshold; CPT: cold pain threshold; HPT: heat pain threshold; PPT: pressure pain threshold; MPT: mechanical pain threshold; WUR: wind-up ratio; LoF/GoF: loss/gain of function. VF16: von Frey weighing 16mN, VF256: von Frey weighing 256mN.

Table S4. Areas under the curve (AUC) of receiver operating characteristic (ROC) analyses.

|  | 1.96SD | | 1SD | |
| --- | --- | --- | --- | --- |
| CTS cohort |  |  |  |  |
| CPT^CST^ | **0.869** | | 0.656 | |
| HPT^CST^ | **0.766** | | 0.616 | |
| PPT^CST^Eraser | **0.797** | | 0.640 | |
| PPT^CST^Thumb | 0.698 | | 0.639 | |
| MPT^CST^ | 0.452 | | 0.551 | |
| NSNAP cohort |  |  |  |  |
| CPT^CST^ | 0.549 | | 0.541 | |
| HPT^CST^ | 0.380 | | 0.466 | |
| PPT^CST^Eraser | 0.409 | | 0.623 | |
| PPT^CST^Thumb | 0.548 | | **0.780** | |
| MPT^CST^ | N/A | | 0.426 | |
| LR cohort |  |  |  |  |
| CPT^CST^ | 0.529 | | 0.493 | |
| HPT^CST^ | 0.500 | | 0.500 | |
| PPT^CST^Eraser | 0.580 | | 0.508 | |
| PPT^CST^Thumb | 0.668 | | 0.594 | |
| MPT^CST^ | N/A | | N/A | |
| Mix cohort |  |  |  |  |
| CPT^CST^ | 0.615 | | 0.575 | |
| HPT^CST^ | 0.465 | | 0.510 | |
| PPT^CST^Eraser | 0.577 | | 0.618 | |
| PPT^CST^Thumb | 0.637 | | 0.682 | |
| MPT^CST^ | 0.529 | | 0.569 | |

Parameters demonstrating acceptable discriminative power (AUC>0.7) are marked in bold. CTS: carpal tunnel syndrome; NSNAP: nonspecific neck and arm pain; LR: lumbar radicular pain/radiculopathy; CPT: cold pain threshold; HPT: heat pain threshold; PPT: pressure pain threshold; MPT: mechanical pain threshold.
